# Indents

**Published:** 1913-11-15

**Authors:** 


### INDENTS.

☞Brief Articles of Technical or Scientific Value will receive notice and comment. Contributions invited.

*Burnishing Gold Inlays.* Inlay margins should not be burnished after the cement has begun to set. If they require burnishing do it before cementing or before the cement begins to set.—J. H. Crossland, in *Dental Brief.*

*Impression Plaster.* To prevent impressions taken with plaster of partial dentures from breaking into too many pieces, mix the plaster with powdered talcum, one part of talcum to three parts plaster. It sets just as well but not so hard and when it breaks it will break in larger pieces. It does not cling to the teeth so tenaciously either. D. W. Barker, in *Dental Summary.*

*Placing Chloropercha in Root Canals.* Thoroughly clean, disinfect and dry the canal, then inject a few drops of wood creasote, or carry it up with a fine broach. Then inject a little chloroform to displace the creasote, then insert a suitable gutta percha point wet with the solution of chloropercha and force it to the end of the root by tapping the plugger gently.—D. J. A. Libbey, in Aug. *Summary.*

*Poisoning from Anilin Dyes in Footwear.* In one case a healthy young man wore a pair of russet shoes which had been dyed black and were still not quite dry. In two hours he became livid, with extreme cyanosis, the lips bluish, the ears purple, the nostrils retracted; the respiration was 29, the pulse, small but regular, was 120 and the skin was chilly, but there was no nausea, dizzines, syncope or tendency to collapse. The urine was blackish but none passed after the first hour. The discovery of the newly blackened shoes in a corner of the room where the young man had tossed them on his return home in the above condition, three hours after he had put them on, cleared up the diagnosis. The symptoms soon subsided. In a second case the young man had put on the newly dyed shoes without socks and started out on his bicycle. In fifteen minutes he was seized with nausea, and fell in a syncope from his wheel. He was picked up in serious collapse with intense cyanosis of the face, respiration 42 , pulse filiform the heart sounds dull. After four hours he passed a blackish urine and in another hour the symptoms began to abate but the weakness persisted for three days, during which period the young man was incapable of the least physical or mental effort. Creyx' third patient was a woman who grew pale and dizzy during the afternoon on several occasions. She went at once to bed each time and the symptoms subsided. She finally discovered that the attacks coincided with the wearing of green stockings, and they never recurred after she had discarded these stockings. In 1910 Landouzy reported ten cases of similar poisoning from russet shoes dyed black, all in children. He experimented on rabbits and found that the intact skin readily absorbs the anilin dyes; the animals died when absorption was through an opening in the skin. Heat favors the absorption. Mongour in 1908 reported a case in which the symptoms from the poisoning proved severe and lasted a long time. Creyx comments on the rarity of such phenomena considering the common use of the anilin dyes. The shoemaker who had dyed the shoes in the first case, he reports stated that he had used the same dye on hundreds of other shoes, in his own family among others, and never a suggestion of trouble had been noted among the other users. The dye consisted of anilin black in anilin oil. The bibliography on the subject is appended.—*Journal A. M. A.*

*Tragacanth Jelly.* Dr. Van Woert in the September *Cosmos* gives the following formula for making what he calls tragacanth jelly; or a solution for mixing porcelain powder so that it will adhere more tenaciously when using the porcelain in making all porcelain carved crowns. To one half ounce of alcohol add one eighth ounce of powdered tragacanth gum and stir thoroughly. The alcohol will not dissolve the gum, but an emulsion will occur, keep stirring and add quickly one and one half ounces of water which will dissolve the gum and produce a smooth jelly like mixture. A proper amount of this tragacanth jelly added to the ground porcelain will make an adhesive mass that can be carved or molded better than if water alone be used as the exsipient.

*Pressure Anesthesia in Multi-rooted Teeth* D. W. N. Miller, in September *Cosmos* gives this method on anesthetizing the root pulps in molar teeth. Anesthetize as much of the pulp as is practicable by high or low pressure method as may be most expedient. Usually the bulbus portion and the larger canal will be effectively done in this way. If so remove the bulb and larger root pulp and dress the root canal with a wisp of cotton covered over with a bit of wax tightly packed but not covering the other canals. Then apply the cocain solution to other canals containing the unanesthetized pulps and by a suitable pressure method they can be fully anesthetized with no harm to the canal from which the pulp has already been removed. The wax and cotton is then removed from the canal by means of a barbed broach.

*Quinine and Urea Hydrochlorid.* The *Therapeutic Gazette* of July 10th calls attention to the delerious effects of this drug when used as a local anesthetic. Cases are cited of extensive sloughing and with difficult healing. In one case an extensive abscess followed the use of a one per cent solution. A two per cent solution caused a temperature of 103 degrees lasting four days, resulting the sixth day in a local abscess. Another case caused a violent discoloration lasting five days with persistent anesthesia for the five days. On the seventh day localized gangrene appeared, with a foul odor, but no suppuration. The gangrenous tissue was surgically removed.—October *Cosmos*.

*Formaldehyd Intoxication.* The Feb. Journal of the A. M. A. Comments on an article on this subject by J. Watt, in *Progress Dentair Paris.* Since Formaldehyd has been so much used in denaturing alcohol and in dental therapeutics many cases of dermatitis have been reported. The dermatitis is persistent and is aggravated by soaps made of animal fats, woolen clothing etc. Soaps made of vegetable oils are agreeable. So many cases are appearing that the author suggests that formaldehyd should not be used at all as a therapeutic remedy; but relegated to the embalmers and pathologists. When a dermatitis appears, wash the affected parts not more than once a day with castile soap, and use olive oil as a cleanser at other times. Ointment of Zinc oxid one part, starch two parts, and petrolatum eight parts may be applied to the affected parts without friction especially during the acute stage, when the cuticle is dry it may be gently rubbed in. Wear light covering, such as loose cotton gloves for the hands if affected, but never rubber gloves. Internally it is as fatal as phenol, acting principally on the nervous system.—October *Cosmos.*

*A View of Anesthesia* Broadly speaking the term "anesthesia" may be used to designate any temporary or reversible lowering or loss of the vital responsiveness, or of the normal automatic vital activity, under the influence of certain artificial substances or conditions. This property of complete reversibility, whereby the normal activities of the organism may completely return when the influence of the anesthetic has passed off, distinguishes it clearly from those irreparable losses of physiologic power which are allied to death. It is only natural that so unique a manifestation should incite investigation of the inherent nature of anesthesia. Consequently there have been various theories of anesthetic action.

The fundamental factor in anesthesia is a loss of what biologists term the irritability of cells or tissues. They no longer exhibit a characteristic response to stimuli to which response is ordinarily obtained. It would lead us too far to take into consideration here the many conditions which are known to alter or abolish the irritability of an organism. Modifications of temperature, certain electrical currents, fatique and mechanical shock are among them. Irritability may be more easily modified, however, by the use of chemical substances than by any other means; and for the medical investigator interest here centers chiefly in a group of largely unrelated substances called anesthetic by the physician. Many of the anesthetics are soluble in fats and fat-like compounds (lipoids) and likewise dissolve them. The Meyer-Overton theory of narcosis propounded a few years ago holds that there is a relation between the lipoid-solvent power of a larger number of organic anesthetics and the intensity of their narcotic power; according to this the chief factor in determining the action is the coefficient of the relative solubility of the anesthetic in water and in lipoids, such as the nervous system contains in great abundance. In other words, for any series of fatsoluble compounds the narcotic action increases as the lipoid solubility increases and the water solubility decreases.

The view whereby this form of anesthetic action depends essentially on a modification of tissue lipoids has received wide-spread attention. There is much left undetermined thereby with respect to the further mechanism of anesthesia and the precise way in which alteration in the lipoid components of cells changes their irritability. Verworn, in particular insist that anesthetics act primarily on the oxidative mechanisms of cells. It is well known that lack of oxygen usually is attended with a decrease in irritability, so that results closely resembling, if not identical with, anesthesia may be produced thereby. It is true that the rate of oxidation in active tissues may be lowered during anethesia; but as Lillie, among others, has emphasized, this effect is rather a consequence than a cause of the lessened activity.[1] He adds that

---

1. Lillie, R. S.: The Physicochemical Condition of Anesthetic Action, Science, June 27, 1913, p. 859.

obviously whenever free oxygen is necessary to the normal activities of a tissue its with-drawal will arrest those activities but the effects produced by lack of oxygen are not to be identified with anesthesia because of such incidental resemblances or parallelisms.

Even for the practical aspects of anesthesia it is of no little moment to establish the fact that tissue asphyxiation or the suppression or prevention of oxidation processes is not the essential basis of anesthesia. How then are we to regard the antistimulating action of anesthetics? How is irritability suppressed by them? A partial explanation of this is the fact, which Dr. Lillie, of the University of Pennsylvania has recently emphasized anew, that the primary process in stimulation is a membrance process. The permeability of cells is modified or determined by membranes. According to Lillie, anesthetics produce their essential effects by modifying the properties of the semipermeable plasma-membranes of the irritable tissues, making these structures more resistant to changes of permeability than normally. Since variations of permeability are essential to stimulation, the irritable tissue is thus rendered temporarily insensitive or irresponsive.

In recording these statements we are not giving any final solution to the varied problems of anesthesia; but the seat of action of anesthetic compounds has been more definitely located. On the view championed by Lillie the membrane is a main controlling factor in cell processes; and by changing its state, whether by lipoid-dissolving anesthetics or otherwise, we may alter the entire physiologic activity of the cell. *Journal A. M. A.* September 24, 1893.

*Inlay Cavity Preparation.*

All cavities should be carried to the angles, we did not mean necessarily that the cavity should be carried to the angles of the tooth. We know that the angles are immune areas, but we sometimes reach immune areas before we reach the angles of the teeth; therefore it depends upon the shape of the tooth, the prominence of the point of contact, etc, as to just where it is necessary to go in order to reach this area. In other words, we want to reach an area that is kept clean by the excursion of food, by the use of the toothbrush, by all methods that clean the surfaces of the teeth, and when we have arrived at that point we have arrived at the point where we want to lay our cavity margin. I fear many of us are basing our opinions of inlays upon too short an experience. We know it takes years of accurate records to make correct deductions. I think many of us are placing inlays in cavities that are practically immune to decay. The trouble people are having at this period of life is a recurrence due to faulty fillings. In a great many instances, we are now practicing "extension for prevention" because it is more convenient to do it in preparing cavities for inlays and some of us, I am sure, are comparing our results with the cavities we prepared some years ago for gold foil fillings, leaving the cavities as small as the conditions would permit. We have gone to the other extreme. We are making cavities as large as necessary, so that we must place the wax and remove it easily.

I would like to call attention again to the point that we must have a lot of accurate records. I doubt if many of us are keeping records from year to year, so that we can go back to them and make accurate comparisons, so that we can draw deductions as to whether these fillings are permanent or not. I am not decrying the gold inlay. There are places and cavities in which no other material will do as well as a gold inlay, but there are several places where a gold filling is far better than a gold inlay can ever be.

A great many believe that an inlay presents a much shorter method of filling a tooth. As a matter of fact, I do not think that is true, but whether it is true or not, that should never be the governing principle as to whether we should use an inlay or gold foil filling or some other filling. It should be the best filling for that individual case, and there is no best filling except for individual cases. You cannot compare a gold foil filling or a gold inlay with amalgam unless you have a definite cavity and definite conditions to deal with. There are lots of mouths in which we cannot place gold filling due to peridental trouble. We cannot subject the patient to malleting. Therefore, a gold inlay will go into these places and will make an excellent filling.—Dr. Gethro, in Sept. *Dental Review*.

*Inlays as Bridge Anchors.*

I am a firm believer in inlays as anchorage for bridges, and I wish to say a few words in their favor. It is true, I have had a good many failures like others, but from these failures I have learned some things.

There has been a good deal said about shallow inlays. I would like to say that might be misleading. An inlay that goes clear down into the pulp chamber in a solid metal cast I do not believe good practice. It has been my experience that if the inlay is thicker at one point than it is at another, it is apt to cause shrinkage on the thin side. In other words, if you have a mesio-occlusal inlay, your occlusal surface should be about the thickness of the mesial surface of that filling; if the occlusal surface is thicker, you are more apt to have cervical shrinkage. I try to get my inlays of the same thickness all the way around even though I have to fill up with cement to get it. Then to have retention I drop a bit distally in that tooth; it must be in solid dentin. If it is not it will not hold.

There is this much about posts I have found a post that has the same gauge in its entire length will hold as much as a post tapered twice its length. If I can get a good fit into good solid dentin, I am sure the inlay is going to hold.

Another point I would like to bring out is with reference to extensions for inlay abutments. Those buccal and lingual margins must be extended, so that there is good clearance between the attachment of the dummy and the filling. The margins must be extended out, so that food in playing over the bridge has a good chance to cleanse the margins. If it does not the margins are going to fail.

I should dislike to give up inlay attachments if I had to devitalize every tooth I wanted to use as an abutment. I know I can get secure abutments in vital teeth if I can get the distal pit, and have it deep enough without exposing the pulp of the tooth and without creating a great deal of pain.

I had a case not long ago where I had to remove a filling on account of it giving pain. In that case my contact was not enough. It was the same preparation. I thought I could knock that thing out with an instrument for knocking out inlays; I hit the tooth two or three taps, and knocked out the whole length of that fine sound tooth in trying to get the inlay out. If I make a filling that will hold as well as that, it will carry its end of the bridge.—Lester Bryant, in *Dental Review.*

*Novrenin.* Novrenin, is the trade name to a preparation of novocaine, adrenalin, and chloretone, which, is now being offered to the dental profession as a non-secret local anesthetic.

Novocain, as is now generally conceded, is easily the best of the many substitutes for cocain that have thus far appeared. It is non-toxic, and causes practically no irritation when applied in solution to the most delicate tissues. Adrenalin prolongs and intensifies novocaine anesthesia. By reason of its vaso-constrictor property it suspends the circulation in the tissues and localizes the action of the anesthetic. Chloretone is anesthetic, sedative and antiseptic. It also acts as a preservative.

The proportions are as recommended for local anesthesia by Dr. Herman Prinz. While intended primarily for hypodermic injection, it is also recommended topically by application to mucous membrane. It should be useful for administration to patients susceptible to the toxic effects of cocain.

*The Contact Point.* I want to emphasize the importance of the contact point by relating a little personal experience. The contact point has been a great source of annoyance to me, not only in the filling of teeth but in my own mouth. Some twenty years ago a very splendid and competent operator put in a gold foil filling in an upper first molar for me, and also one in the second bicuspid. By some force or stress I broke off the inner cusp of the second bicuspid, and the filling was dislodged in that tooth, and I remember calling on one of my friends to restore the space with a crown. In making a crown for that space he could not restore the contact point which had never been there in those two fillings that had previously been inserted. But the crown was put on, and I went down to the Illinois State Dental Society at Springfield, and if there was anything that was annoying it was the interproximal space between those teeth. I was standing in the lobby with a silk thread trying to dislodge the food from the interproximal space. The dentist who inserted the crown came along and asked what I was doing, and I said, "you have ruined my mouth." He said: "I will fix that for you as soon as we get back to Chicago." He took the crown off, and made a contact point and replaced it, but he did not do it soon enough, so I have lost all the gum tissue in the interproximal space, and I am nearly in as bad condition as I was before, but if the two fillings had had proper contact points in the beginning there would have been no trouble.—G. W. Cook, in *Review*.

